# DNA hypermethylation of alternatively spliced and repeat sequences in humans

**DOI:** 10.1007/s00438-012-0703-y

**Published:** 2012-06-28

**Authors:** Andigoni Malousi, Sofia Kouidou

**Affiliations:** Department of Biological Chemistry, School of Medicine, Aristotle University of Thessaloniki, 54124 Thessaloníki, Greece

**Keywords:** Methylation, Splice sites, Alternative splicing, SINEs, Alus, MIRs

## Abstract

DNA methylation is presently accepted as a tentative regulatory parameter in splicing. Recently, we reported significant methylation differences among various exonic splicing-enhancing elements and alternative splicing events, based on CpG methylation data from the Human Epigenome Project for chromosomes 6, 20 and 22. Presently, using a different computational approach and the same database, we report: (a) significant increase of hypermethylation in intronic and exonic sequences close to acceptor sites, relative to overall introns and exons, respectively (1,973 CpGs examined); (b) frequent CpGs, mostly hypomethylated, in donors and infrequent CpGs mostly hypermethylated, in acceptors; and (c) hypermethylation in cassette exons which are occasionally spliced and have weaker average splicing potential, relative to constitutive exons (*p* < 0.0001). CpGs are hypomethylated in non-coding exons (only 16 % hypermethylation). Single-exon genes, similarly to first exons, frequently contain hypomethylated CpGs, while in internal and last exons CpGs are more frequently hypermethylated. Methylation is also more frequent in strange introns and splice sites processed by the minor spliceosome, e.g., ATAC, (*p* < 0.0001 in all cases), but not in sites of incomplete processing, e.g., retained introns or bleeding exons, (*p* = 0.706 and *p* = 0.313, respectively). Most Alus, which are known to contribute to transcript presentation, are heavily methylated, in contrast with other Alus, e.g., AluJo and mammalian interspersed repetitive elements which have been previously associated with alternative expression. These results elucidate the role of intragenic methylation in association with alternative splicing and facilitate the evaluation of genomic variations/polymorphisms and the development of tools for the prediction of alternative splicing events.

## Introduction

Although epigenetic mechanisms are actively investigated at present, our understanding of the impact of non-promoter DNA methylation is still very limited. Recently, it has become increasingly evident that DNA methylation might be a key parameter in the process of splicing (Luco et al. 2011). This novel role for DNA methylation, which is based on the finding that splicing is a co-transcriptional process (Pandya-Jones and Black 2009; Tilgner and Guigo 2010), is expected to provide a better understanding of the splicing mechanisms and differentiation of gene expression (Pandya-Jones and Black 2009).

In fact, the possible involvement of DNA methylation in the regulation of the splicing process has been recently addressed. Specifically, the studies of Chodavarapu et al. on *Arabidopsis thaliana* reveal that DNA methylation is highly enriched in exons and nucleosome positioning might play a significant role in DNA methylation (Chodavarapu et al. 2010), which, in turn, is associated with the selection of intron–exon boundaries (Schwartz and Ast 2010; Tilgner and Guigo 2010; Choi 2010). Transitions in the methylation state correlating with genomic landmarks such as intron–exon junctions were reported by Hodges et al. (2009) based on single molecule human DNA studies. Finally, the role of DNA methylation in alternative splicing is supported by the recent work of Shukla et al. (2011) in CD45 cells. In this work, the authors showed that the activity of the CCCTC-binding factor (CTCF), which can promote inclusion of weak upstream exons by mediating local RNA polymerase II pausing, can be reciprocated by DNA methylation in a specific exon.

Correlation of the CpG density/methylation state with expression in alternatively spliced sequences is therefore critical for elucidating the epigenetic regulatory mechanisms in splicing. CpG-dense motifs (Malousi et al. 2008) and specific Alu repeats, e.g., Alujb, AluSx and AluJo (Sorek et al. 2002, 2004) have been strongly associated with alternative splicing. In addition, the latter have been proposed to cause frame-shifting or premature termination codons, reduced expression frequencies (Lin et al. 2008) in different pathological conditions, such as X-linked dilated cardiomyopathy (Rimessi et al. 2010), ATM (Pastor et al. 2009) and other human diseases (Vorechovsky 2010). Similarly, mammalian interspersed repeats (MIRs) have been associated with frequently diverse, translational regulation (Huh et al. 2008; Lin et al. 2008).

These observations are in support of a new model for the regulation of splicing, in which DNA methylation plays an important role in constitutive and alternative splicing. More specifically, chromatin remodeling via chromatin-binding proteins such as CTCF, or other protein factors appears to be also related to polymerase stalling, which in turn, is necessary for transcriptional elongation and splicing (Tao et al. 2010; Shukla et al. 2011; Alexander et al. 2010; Oberdoerffer 2012). In addition, nucleosomal positioning, which has been associated with polymerase activity regulation, is reported to be preferentially associated with methylated sequences (Chodavarapu et al. 2010; Shukla et al. 2011). This data, which combines nucleosomal positioning and polymerase activity with splicing efficiency and DNA methylation reveals an immersing role for DNA methylation, related to splice site selection, and possibly alternative splicing. Such a model would in turn provide a better interpretation of disease-related mutations and polymorphism selection (Karambataki et al. 2010). However, to date, there is little direct information from large-scale data on the association of methylation with specific alternative splicing types, splice sites and short interspersed nuclear elements (SINEs), as well as the distribution of methylation in splice sites.

A recent analysis (Anastasiadou et al. 2011) based on the methylation data obtained for 33,352 CpG sequences from different human tissues reported by the Human Epigenome Project (HEP), showed that DNA methylation is significantly associated with alternative splicing events and varies among different categories of exonic motifs (exonic splicing enhancers), which play a regulatory role in splicing. Using a different computational procedure and the same raw data from a variety of tissues, we presently investigated CpG methylation in introns, splice sites, and different SINEs. Moreover, we examined its presence with respect to different forms of alternative splicing events. Our present results provide considerable insight on the association of methylation with splicing selection and promote our understanding of alternative splicing.

## Materials and methods

The present study is based on computational analyses of DNA methylation data from HEP (Rakyan et al. 2004; Eckhardt et al. 2006). HEP is the first systematic and large-scale study of DNA methylation on the human genome that aims to map DNA methylation sites through the 30,000 gene loci. Specifically, we used the 26th release of the HEP pilot data (June 2006) containing the methylation status of 1.88 million CpG sites that were obtained from the analysis of 2,524 amplicons of human chromosomes 6, 20 and 22. In order to examine potential co-localization of the analyzed CpG sites and regulatory elements affecting gene expression, we mapped genomic regions of interest (hg16 genome assembly, July 2003), e.g., transcribed sites, repeats and alternative splicing events to the available methylation sites together with the assigned average methylation scores from 12 tissues ranging from 0 to 100 % for each record. CpG sites with known data that was not further analyzed by the HEP Project and data with no available methylation score was ignored.

We classified the resulted 33,352 CpGs with recorded methylation score, including zero methylation, in three categories depending on their average methylation values. CpG sites with methylation score greater or equal to 80 % (9,012 sites) belong to the dataset of the hypermethylated cytosines (HmC), while CpGs with methylation scores ranging from zero up to 20 % (15,296 sites) are characterized as hypomethylated or low-methylated (LmC). The remaining 9,044 methylation data with methylation levels between 20 and 80 % correspond to a third category containing data with intermediate methylation scores (ImC). This classification has been also applied in other studies and can safely distinguish sequences containing HmC and LmC (Rakyan et al. 2004; Eckhardt et al. 2006).

In the analysis of the 60-nt sequences centered at the splice junctions that contain HmC, ImC or LmC we extracted all hg16 introns, rather than exons, and retrieved their flanking sequences in order to avoid the selection of splice sites preceding and following first and last exons, respectively. Introns and exons of less than 30 nt were excluded in this study in order to avoid overlaps between consecutive splice sequences. Similarly, in the analysis of the 130 nt intronic sequences flanking donor and acceptor sites, overlapping sequences were excluded. The UCSC Table Browser (Karolchik et al. 2004) through the Galaxy platform (Goecks et al. 2010) was used to extract the coordinates and the sequences of the examined regions and the Galaxy’s lift-over tool was used to map genomic coordinates between different assemblies, where needed.

The set of 27,866 constitutive exons is a compilation of all hg16 reference exons subtracted by the 8,735 alternative splicing events that were extracted from the UCSC altEvents track of the same human genome assembly. In addition, the Repeatmasker track of the UCSC Table Browser (Smit AFA 1996–2007) was used to screen annotated SINEs in chromosomes 6, 20 and 22 (hg16 human genome assembly). Finally, for the combined analysis of the methylation and genomic data we developed Perl scripts and for the statistical evaluation of the identified associations we employed Chi-square and *t* tests.

## Results

### Methylation in transcribed sequences of chromosomes 6, 20 and 22

**Fig. 1:**
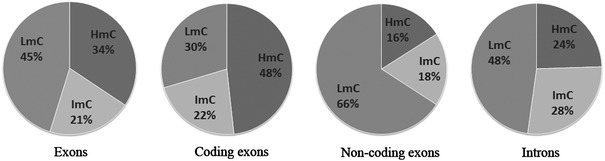
Methylation frequencies (obtained from data in Table 1) in exons, coding exons, non-coding exons, and introns

The proposed computational approach was first applied on the adjusted HEP data in order to measure the distribution of methylation in exons compared to introns (Table 1). Exonic CpGs were divided in two categories: coding and non-coding. The latter are mostly positioned at both ends of the transcribed sequences and play a regulatory role in expression (Lilischkis et al. 2001; Medvedeva et al. 2010). This analysis reveals, as expected (Hodges et al. 2009; Chodavarapu et al. 2010), significant hypermethylation (HmC, 48 %) among coding exons, while CpG-containing sequences in introns are less frequently hypermethylated (HmC, 24 %; Fig. 1). It should be noted, however, that since all presently examined intronic and exonic sequences are CpG-containing, while introns also contain extended CpG-free sequences, the actual intronic methylation frequency is probably lower than that presently shown in Table 1. The results in Table 1 also show that a substantial number of the examined methylated CpGs are included in non-coding exons, and the size of this sample is suitable for valid statistical comparisons. In this case, CpGs in non-coding exons are scarcely hypermethylated (HmC, 16 %; Fig. 1).

**Table 1:** Number of methylated CpGs in selected sequences depending on their coding potential

	Total CpGs	CpGs in exons	CpGs in coding exons	CpGs in non-coding exons^a^	CpGs in introns
HmC	9,012	3,644	2,915	729	2,844
ImC	9,044	2,167	1,336	831	3,219
LmC	15,296	4,795	1,788	3,007	5,553
Total	33,352	10,606	6,039	4,567	11,616

Multiple CpGs may be located in a single sequence

^a^The number of non-coding exons resulted by subtracting the coding exons from exons

**Fig. 2:**
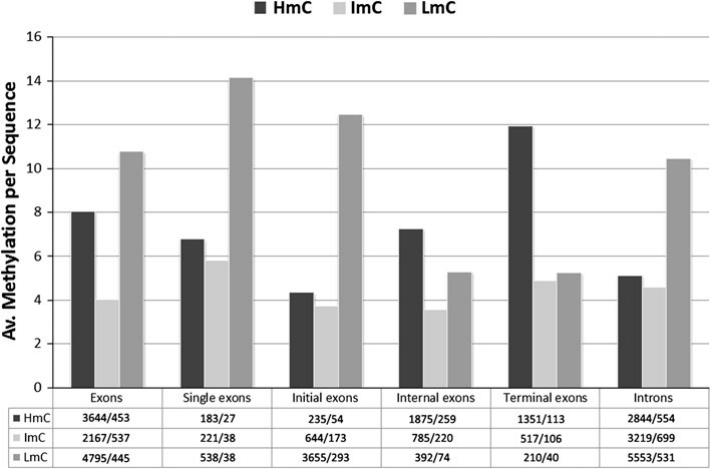
Average methylation per sequence, i.e., the number of identified HmC, ImC and LmC versus the number of analyzed sequences (no. of CpGs/no. of seqs), in total, single, initial, internal and terminal exons and in introns, containing at least one recorded CpG. The number of CpGs per the number of sequences is shown in the embedded table

We then analyzed the frequencies of hypermethylated, occasionally methylated and hypomethylated CpGs (HmC, ImC and LmC, respectively), in different exonic and intronic sequences. For this purpose, the CpGs of each of the above categories were divided by the corresponding number of non-duplicated sequences containing at least one CpG in the corresponding category (Fig. 2). In addition, exonic sequences were classified as initial, terminal, single and internal exons. Initial and terminal exons (mostly non-coding) are more CpG-dense compared to internal (coding) exons (total number of CpGs per number of sequences: 20.55 for initial, 22.08 for terminal exons, 16.10 for internal, and 26.75 for single exons), while initial exons are also more hypomethylated compared to intronic sequences.

The hypermethylation frequency in initial exons is comparable to that in introns (Fig. 2). Single exons have common epigenetic characteristics both with initial and partially with internal exons. Specifically, the average number of hypermethylated CpGs per sequence is similar in single and internal exons, but single, similarly to initial exons, also have higher LmC frequencies and are more CpG-dense than other exons. In addition, they share similar average numbers of CpG sites per sequence of variable methylation with terminal exons.

Overall, the data in Table 1 and Fig. 2 show that, in addition to dense exonic versus poor intronic methylation, limited methylation close to the transcription start site is probably required to establish the transcription process, while high CpG density is necessary to block transcription initiation or to terminate it (in single exons and in terminal exons, respectively), when required. Furthermore, this data might also indicate that high, possibly specific methylation, is required to regulate transcription of single, initial, terminal exons. In internal exons, where the average hypermethylation per sequence is high, though considerably lower than in terminal exons the CpG density is lower possibly introducing a transient halt during transcription. In this case the presence of methylation might be also associated with the spliceosome formation (Oberdoerffer 2012; Anastasiadou et al. 2011).

### Distribution of methylation in donors and acceptors

**Fig. 3:**
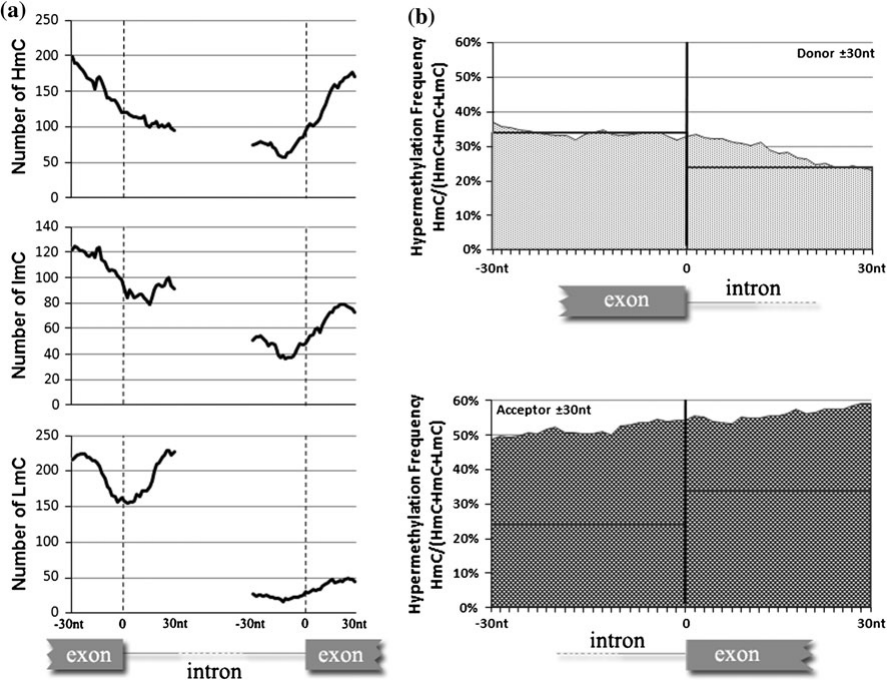
**a** Distribution of methylation at both sides of donors and acceptors in 20-nt sliding windows of 60 nt total length centered at the splice junctions. The exon–intron boundaries are shown in vertical *dashed lines.* **b** Hypermethylation frequency corresponding to the same region shown in **a**. The average HmC frequency in whole exons and introns is shown in *horizontal lines* (obtained from Fig. 1)

The distribution of methylation and the CpG frequencies in splice sites were evaluated with respect to the position of each analyzed CpG relative to the intron/exon boundaries. These sites have been previously shown to be associated with distinct methylation transitions in single cell experiments (Hodges et al. 2009). Figure 3a illustrates the distribution for each methylation level in donors and acceptors in 20 nt sliding windows. In total, 1,973 CpGs (763 HmC, 491 ImC and 719 LmC) were identified in 60 nt regions centered at splice junctions. At the splice donor sequence corresponding to the U1snRNP binding site (Roca et al. 2005) the LmC frequency exhibits a local minimum compared to the HmC (*p* = 0). In addition, the frequency of methylated cytosines (HmC and ImC) in the intronic region close to acceptors is also very low compared to the intronic sites close to donors, indicating that, CpGs in donors are frequently hypomethylated contrary to those in the corresponding acceptor region (*p* = 0).

The hypermethylation frequency, i.e., the number of HmC divided by the total CpGs, either HmC, ImC or LmC identified in each region is shown in Fig. 3b. Close to the splice junction, the hypermethylation frequency is significantly higher at the acceptor site (both intronic and exonic) compared to the average frequency in introns or exons (Fig. 1). In donors, the hypermethylation frequency is comparable to the average frequency in introns and exons. This data reveal that, although CpGs might be frequent in splice donors, only a small fraction is hypermethylated, while the limited number of CpGs present in acceptors is mostly hypermethylated. Thus, in 5′ intron ends there is probably a strict selection of CpGs undergoing methylation, while hypermethylation is very frequent in 3′ intron ends compared to all other sites examined.

**Fig. 4:**
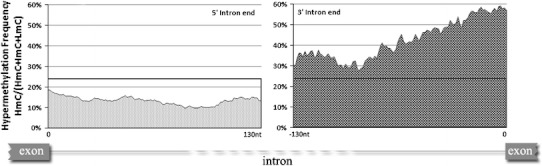
Hypermethylation frequency in the 130-nt intronic sequences flanking donor (*left*) and acceptor (*right*) sites, using 20-nt sliding windows. The average HmC frequency in introns, obtained from Fig. 1, is shown in *horizontal lines*

We then analyzed the hypermethylation frequencies among CpGs in the intronic sequences, flanking splice junctions using 20-nt sliding windows (Fig. 4). Up to 90 nt from the acceptor junction, the hypermethylation frequency decreases but remains significantly higher compared to the average intronic, or even to the corresponding donor sites. Approximately 100 nt from the acceptor site the intronic hypermethylation frequency decreases. Extending by 30 nt this region it is evident that the HmC frequency becomes less variable and it remains relatively low. At the donor site the hypermethylation frequency extended in the 130-nt intronic region is less variable than the corresponding frequency at the acceptor site and also continuously lower than the average frequency of introns.

In addition, the methylation density in the 100-nt intronic region close to splice junctions was found to be comparable to that in exons and approximately 100-fold higher than the corresponding density in total intronic sequences shown in Table 2. The density of the LmC and partly methylated CpGs (ImC) close to the splice junctions is very high compared to introns. On the contrary, the hypermethylated CpG density is very low in donors (Table 2). Thus, in 5′ intron ends close to splice junctions a strict selection of CpGs undergoing methylation, extends for at least 100 nt within introns, while the probability of hypermethylation within acceptor CpGs is higher than in any other site presented in Table 1.

**Table 2:** Methylation density in different sequences

	5′ intron ends (100 nt)	3′ intron ends (100 nt)	Introns	Cassette exons	Constitutive exons
HmC	0.024	0.022	0.0003	0.0259	0.0095
ImC	0.021	0.018	0.0002	0.0134	0.0054
LmC	0.059	0.045	0.0005	0.0153	0.0167

Total CpGs/(number of non-duplicated sequences containing at least one CpG × average length of the sequences)

Provided that previous studies based on a single cell analysis (Hodges et al. 2009) have shown sharp methylation changes in intron/exon boundaries, it is conceivable that the presently shown high methylation scores and slow demethylation transitions in intron/exon boundaries, are related to the fact that this analysis is based on average methylation frequencies for all tissues and cells. Together, these results show that methylation in the 100-nt edges of intronic sequences is probably very significant and selective for determining the “epitype” (Hodges et al. 2009), i.e., epigenetic regulation and splice site selection in individual tissues.

### Methylation and alternative splicing

#### Methylation frequencies in cassette and constitutive exons

We then tested whether the methylation frequency might be altered in association with alternatively spliced sequences such as cassette exons, exons with weak, non-canonical splice sites, or in splice sites processed by the minor spliceosome. For this purpose, sequences containing CpGs with recorded methylation data were further analyzed for their presence in alternatively spliced forms. The most frequently observed alternative splicing events involve cassette exons which exhibit well-defined intron-exon boundaries and are characterized by lower splicing potential (scores) compared to constitutive exons (Clark and Thanaraj 2002; Garg and Green 2007).

As shown in Table 2, the density of hypomethylated CpGs is higher in the 990 analyzed constitutive exons (1.67 % LmC vs. 0.95 and 0.54 % for HmC and ImC, respectively), while the hypermethylation density is higher in cassette (64 in total) than constitutive exons (2.59 vs. 0.95 %, respectively). Thus, cassette exons are characterized by a considerable increase of the methylation density, while their total CpG density does not significantly vary from that of constitutive exons. Although the sample of cassette LmC is limited, the methylation differences of HmC and LmC between total exons and cassette sequences are very significant (*p* < 0.0001; Table 3).

**Table 3:** Statistical analysis of hypermethylation differences (*p* values) between total exons, introns relative to alternative splicing events

	Total exons		Total introns
Cassette exons	*p* < 0.0001	Retained introns	*p* = 0.706
Bleeding exons	*p* = 0.313	ATAC introns	*p* < 0.0001
		Strange introns	*p* < 0.0001

**Fig. 5:**
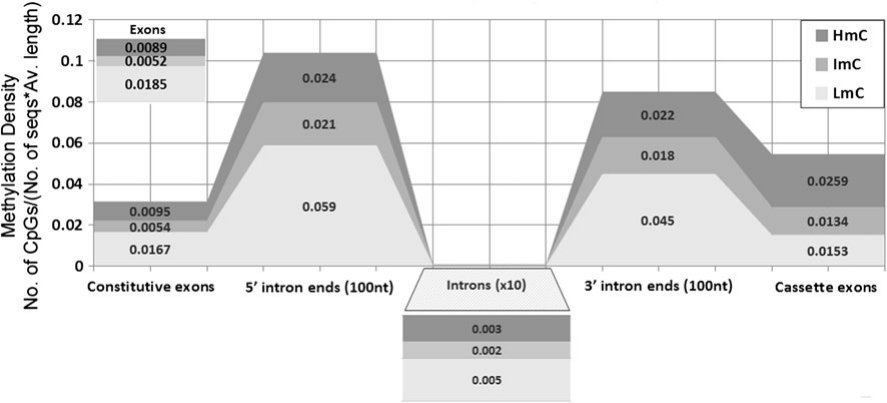
Graphical representation of methylation densities in exons (cassette, constitutive and total), introns and 100 nt 5′, 3′ intronic sites. The relative widths of the *different grey tones* correspond to methylation frequencies. The shown frequency scale is enlarged 10× for introns

The above findings (summarized in Fig. 5) reveal that significant methylation differences are observed between alternative and constitutive exons depending on the type of alternative splicing process. Particularly prominent is also the “infiltration of methylation” in intronic sequences neighboring splice junctions and the methylation differences between donors and acceptors. Information regarding alternative splicing in association with the distribution of methylation in exonic splicing enhancers (Anastasiadou et al. 2011) will facilitate the design of improved computational tools for alternative splice site prediction (Richard et al. 2010; Wang and Marin 2006) and evaluation of disease-related mutations and polymorphisms (Kouidou et al. 2009).

#### Splicing potential in cassette and constitutive exons

The flanking 5′ and 3′ intron ends of the analyzed cassette and constitutive exons were then evaluated for their compliance with the canonical forms, i.e., GT dinucleotides for the following donors and AG for the preceding acceptors as regards to their methylation and their splicing strength. The flanking intron ends were identified in cassette and constitutive exons using UCSC altEvents track in our sample and the splicing potential of canonical splice sites was calculated using the identification method proposed by Shapiro and Senapathy (1987).

Using this approach we observed that 63 out of 64 cassette exons containing at least one HmC, ImC or LmC were canonical (overlaps included), showing that the presently examined cassette exons comply with the canonical splicing forms (*p* < 0.0001). The only cassette exon with non-canonical splice sites flanks a GC donor and AG acceptor. Of the 990 identified constitutive exons that contain at least one HmC, ImC or LmC (Table 4), a substantial number corresponds to first and last exons. Almost all internal constitutive exons exhibit canonical AG/GT intron ends (99.34 %). As expected from the results shown in Fig. 2, we also observed that hypermethylation is frequent in terminal and less prominent in initial exons, while the opposite is found for rarely methylated CpGs (LmC). However, intermediate methylation was equally frequent in initial and terminal exons. Among constitutive acceptors, methylation differences (HmC/ImC and HmC/LmC) are statistically significant, and the same is observed in some cases in donors (HmC/LmC). However, in donors we also observed that there is no significant difference between the frequency of hypermethylated CpGs (HmC) and ImCs, i.e., CpG cytosines which are either methylated only in specific tissues, or at specific developmental stages. This finding probably indicates that in donors and acceptors the methylation process is regulated by different parameters. All other differences are statistically significant. As expected from the results shown in Fig. 2, in constitutive terminal exons hypermethylation is more frequent relative to initial exons while the opposite is found for rarely methylated CpGs (LmC). Intermediate methylation was equally frequent in initial and terminal exons.

**Table 4:**
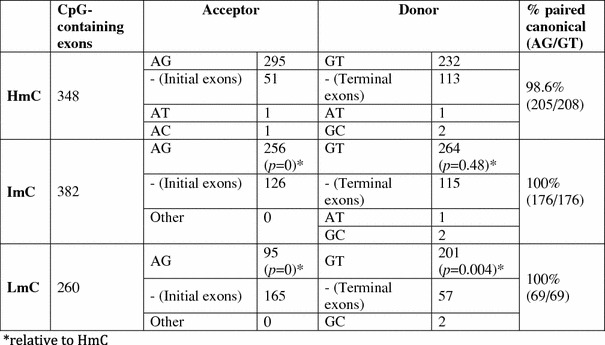
Analysis of splice sites flanking constitutive exons (including initial and terminal exons) which contain at least one HmC, ImC or LmC

“Other” refers to non-canonical donors and acceptors of internal exons unless otherwise specified. Paired canonical: Exons flanked by canonical splice sites (both acceptor and donor)

* Relative to HmC

The splicing scores of the examined donors and acceptors also differ between cassette and constitutive exons. Acceptors with canonical form that precede cassette exons exhibit lower splicing scores compared to constitutive exons (Table 5). The data on cassette exons are in agreement with previous observations by Choi (2010), which reveal that methylation is enriched in exons with weak splice sites and is probably associated with spliceosome positioning and with previous findings for cassette exons (Garg and Green 2007; Clark and Thanaraj 2002). These data are in support of a splicing-enhancing role for exons with weak splice sites.

**Table 5:** Splice site scores and average scores of the three categories for cassette and constitutive exons examined (all canonical, containing CpGs)

	Cassette	Constitutive
Donors		
HmC	78.53 (33)	82.71 (232)
ImC	80.04 (26)	83.44 (264)
LmC	77.84 (4)	83.86 (201)
Av. score	78.80	83.34
Acceptors		
HmC	48.95 (34)	54.03 (295)
ImC	49.99 (26)	54.32 (256)
LmC	46.16 (4)	54.31 (95)
Av. score	48.37	54.22

The corresponding number of exons is shown in parenthesis

#### Methylation in other alternatively expressed loci

**Fig. 6:**
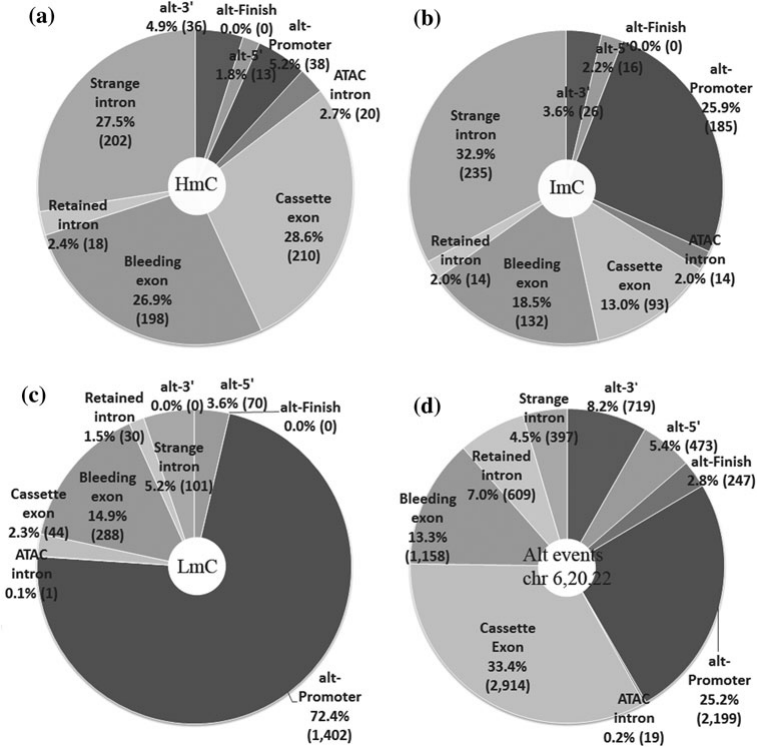
Distribution of the studied **a** HmC, **b** ImC and **c** LmC with respect to their co-occurrence with alternative splicing events. The frequency of all reported alternatively spliced introns and exons in chromosomes 6, 20 and 22 is shown in **d**

In order to further verify the association of methylation with various, less frequent types of alternatively spliced sequences (other than cassette exons), we identified the alternative splicing events among the analyzed CpG-containing sequences studied by HEP (hg16, July 2003) and correlated them with the methylation status in these sequences. Specifically, each methylation locus was searched through the list of annotated alternative splicing events. Figure 6 shows the matched events per methylation category.

The results in Fig. 6 reveal that most significant differences in methylation are observed in specific types of alternative splicing events, e.g., in alternative promoters, in alternative 3′ ends, and in strange introns and bleeding exons. Specifically, Fig. 6 shows that hypermethylation is more frequent among alternatively spliced sequences in which the intron-exon boundaries are well defined, relative to other splicing events defined as “bleeding” in which the intron-exon boundaries are not well defined. Thus, bleeding exons are less hypermethylated compared to cassette exons (32.04 % HmC, vs. 60.52 % in cassette exons) or compared to the average coding exons (48 %, Table 1). In addition, other types of sequences resulting from splicing events which are considered products of partial spliceosomal processing, e.g., retained introns, are characterized by low methylation levels (HmC, 29.03 %; LmC, 48.39 %). In both bleeding exons and retained introns the statistical difference with total exons and introns is not significant (*p* = 0.313 and *p* = 0.706, respectively; Table 3). In addition, strange introns are significantly more hypermethylated than total introns (Table 1; Fig. 6) and the same is observed with ATAC introns, a specific intron category with non-canonical intron ends, which are processed by the minor spliceosome (HmC, 57.14 %, similar to the frequency of cassette exons), indicating that there might be a significant correlation of methylation frequencies and selection of different intron ends.

Once more, this type of analysis is in agreement with the results shown in Table 1 and Fig. 2 and reinforces the finding that methylation is significantly correlated with the selection of expressed sequences and acts, in a sequence-specific, orchestrated manner, depending on the splice site acceptor and donor sequences (Tables 4, 5; Figs. 5, 6). In contrast, “deviations” occurring by incomplete processing of one exon boundary, i.e., bleeding exons, are associated with lower methylation frequencies and other selection processes. All previously described differences are very significant (*p* values are shown in Table 3).

### Methylation in SINEs

**Fig. 7:**
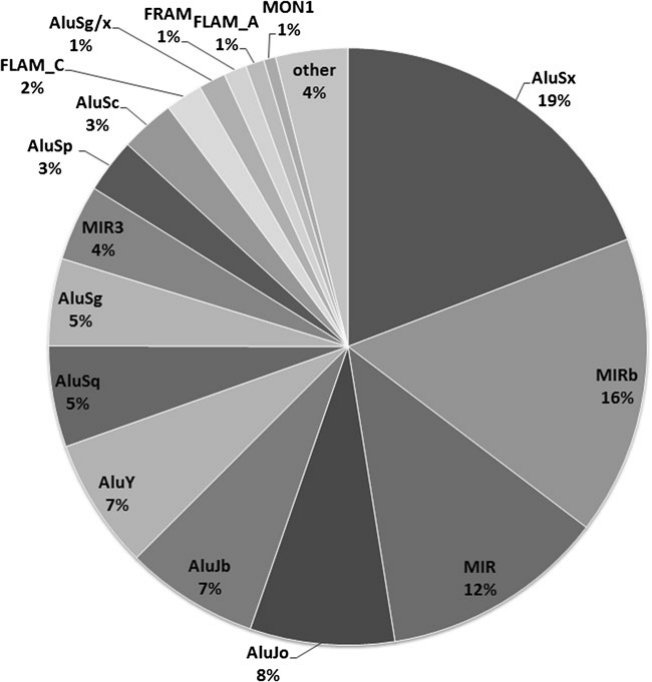
Relative frequencies of different types of SINEs in chromosomes 6, 20 and 22. Some Alus (AluSq/x, AluSg1, Alu, FAM, AluYd2, AluYc2, AluJ/FLAM, and AluYd3a1) generally denoted as ‘other’ have frequency greater than 0.3 % (not shown)

The frequency of methylation in SINEs, which are also known regulatory parameters for transcript inclusion, was then evaluated using RepeatMasker (Smit AFA 1996–2007) in order to identify methylation differences which might discriminate the various SINEs relative to their epigenetic modification state. These differences were then compared to the previously described impact on transcript inclusion for the different SINEs. We identified 308 SINEs containing HmC, ImC or LmC among the 167,304 detected SINEs in chromosomes 6, 20 and 22 (hg16, July 2003). The identified SINEs correspond to 0.184 % of the total SINEs in these chromosomes, regardless of their CpG content (Table 6). Analysis of the different SINE categories (Fig. 7) reveals a variety of different Alus present in this sample. Among these, AluSx, MIR and MIRb are most frequent.

**Table 6:** Total CpGs observed in SINEs and the number of SINEs containing at least one HmC, ImC and LmC

	Total CpGs in SINEs	SINEs containing at least one CpG (CpGs per SINE)
HmC	169	55 (3.07)
ImC	89	51 (1.74)
LmC	50	23 (2.17)
Total	308	129 (2.38)

The average number of CpGs per SINE sequence is shown in parenthesis

**Fig. 8:**
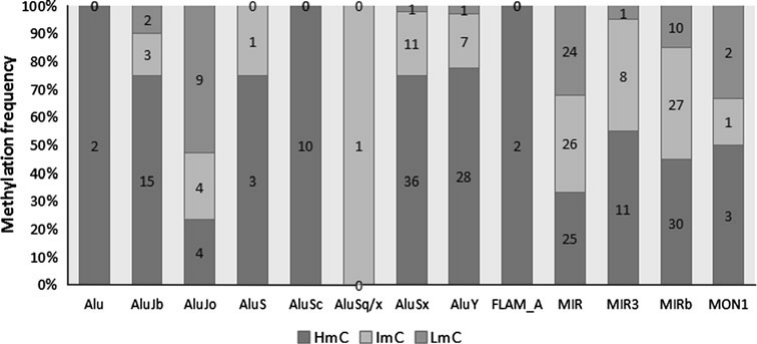
Distribution of methylated CpGs among different types of SINEs. The total number of SINEs in chromosomes 6, 20 and 22 is 167,304

Most of these SINEs are hypermethylated (HmC, 54.87 %; Table 6), but strong differences of the methylation frequencies among Alus and particularly MIRs (Fig. 8) are also observed. While most Alus, including AluSx, which is the most frequent Alu present in this sample and in chromosomes 6, 20 and 22 (Fig. 7), are rarely hypomethylated, MIRs (MIR, MIR3 and MIRb), as well as AluJo are characterized by lower hypermethylation frequencies (Fig. 8). The difference of hypermethylation between the above repeat sequences, i.e., AluJo and MIRs, relative to all the remaining SINEs is statistically significant (*p* < 0.0001). Methylation among MIRs also varies. In view of these significant variations of the methylation frequencies in AluJo and MIRs which have been previously associated with alternative splicing (Lin et al. 2008; Shen et al. 2011) and transcript inclusion in coding sequences, it is probable that methylation might also contribute to the regulation of “exonization” in SINE-containing methylated sequences.

## Discussion

Contrary to promoter methylation, the role of exonic and intronic methylation is still ill-defined. Recent evidence points out the association of promoter methylation with chromatin modification, transcriptional elongation and RNA polymerase stalling (Chen et al. 2011). This data also indicates a similar role for intragenic methylation in splicing and its regulation. Our data provides a detailed description of the distribution of methylation and of epigenetically unmodified CpGs in different human sequences with respect to splicing, “weak” and “strong”, alternative and constitutive splice sites, and “exonization” (single, initial and terminal exons, as well as Alus which are known to contribute to “exonization”). In addition, we provide a detailed account of sites, which are partially or rarely epigenetically modified. Based on our results, it becomes evident that the cytosine methylation density is modified according to the transcriptional and splicing potential of a sequence, and could thus act as a supplementary parameter for regulating the splicing strength of splice sites (in defining “spliceable” sequences). Moreover, it becomes evident that different “rules” regulate the methylation frequency in donors and acceptors, as well as at the 5′, relative to the 3′ ends of genes. More specifically, our results reveal that intragenic methylation is strongly associated with transcribed regions in which specific transcript processing is required, such as alternative exons, weak acceptors, as well as rare ATAC introns. Epigenetic marks, such a reduction of methylation appear to define exon/intron boundaries. It should be noted that in a previous publication we showed an increase of methylation in nucleosome-binding consensus sequences, which has also recently been shown to regulate the splicing efficiency (Anastasiadou et al. 2011).

During the preparation of this manuscript, two studies on genome-wide methylation in rat (Sati et al. 2012), as well as in specific cell types have been reported (Zhou et al. 2012). Although different analytical procedures have been applied in these studies and the results are not directly comparable, they both emphasize the role of DNA methylation in the alternative transcript inclusion. Our study presents a detailed account of the average methylation in more than one human tissue as well as the average degree of methylation (hyper-, inter-, hypomethylation) for each CpG site in different developmental stages. In conclusion, the above data are critical for understanding the role of the vast majority of DNA methylation, which is focused in intragenic regions. It is, however, conceivable that methylation involving, but yet undefined regulatory regions related to miRNA coding and other regulatory sites of the genome is expected to play significant role in expression and differentiation.

Moreover, we analyzed data with respect to the CpG frequency, i.e., the number of methylation targets for each sequence. Previous studies (Davey and Allan 2003; Dhasarathy and Wade 2008) have shown that the presence of CpG sequences alters the DNA conformation and nucleosome binding independently of the methylation density. Our detailed analysis indicates that the CpG and methylation densities might act as independent parameters defining the selection and efficiency of splice sites and promoters, and the transcript inclusion of exons or intronic sequences. In UTRs and splice donors, where transcriptional stalling is required, the appropriate protein assembly is probably dependent on the very frequent presence of methylation and the recognition by different methyl-binding proteins (Dhasarathy and Wade 2008). Similarly, methylation might facilitate splicing of weak splice sites and alternatively spliced exons by assisting spliceosome recruitment. In promoters and first exons, high CpG density probably denotes the presence of a tentative regulatory target, while their accessibility is regulated by methylation. In introns where splice sites and intronic acceptors which are CpG-poor, the chromatin architecture, looping and absence of nucleosomes, probably facilitates spliceosome binding and splicing. Finally, the expression of intronic CpG-dense sites, such as Alus, might be dependent on their methylation density. Thus, analysis of the CpG density relative to the methylation distribution presented in this study contributes to a better understanding of a new role of DNA methylation in transcription and introduces new constituents to the process of alternative splicing.

Comprehensive analysis of all available data and large-scale studies on epigenetic modifications, are now required to promote our understanding of the epigenetic regulation of splicing and transcript inclusion. However, since DNA imprinting includes several forms of epigenetic modification, such as non-CpG methylation, also frequent in splice sites (Shen et al. 2011), and other forms of cytosine modification, e.g., hydroxymethylcytosine, also presently considered critical for DNA expression (Pastor et al. 2011), it is evident that the present data provides only “quick glances” at the highly complex epigenetic code and its impact on expression.
